# Measuring depression after spinal cord injury: Development and psychometric characteristics of the SCI-QOL Depression item bank and linkage with PHQ-9

**DOI:** 10.1179/2045772315Y.0000000020

**Published:** 2015-05

**Authors:** David S. Tulsky, Pamela A. Kisala, Claire Z. Kalpakjian, Charles H. Bombardier, Ryan T. Pohlig, Allen W. Heinemann, Adam Carle, Seung W. Choi

**Affiliations:** 1 Department of Physical Therapy, University of Delaware College of Health Sciences, Newark, DE, USA; 2 Kessler Foundation Research Center, West Orange, NJ, USA; 3 Department of Physical Medicine and Rehabilitation, University of Michigan Medical School, Ann Arbor, MI, USA; 4 Department of Rehabilitation Medicine, University of Washington School of Medicine, Seattle, WA, USA; 5 Department of Physical Medicine and Rehabilitation, Feinberg School of Medicine, Northwestern University and Center for Rehabilitation Outcomes Research, Rehabilitation Institute of Chicago, Chicago, IL, USA; 6 Cincinnati Children's Hospital, Cincinnati, OH, USA; 7 McGraw-Hill Education CTB, Monterey, CA, USA

**Keywords:** Health-Related Quality of Life, Outcomes Assessment (Healthcare), Patient Reported Outcomes, Depression, Spinal Cord Injuries

## Abstract

**Objective:**

To develop a calibrated spinal cord injury-quality of life (SCI-QOL) item bank, computer adaptive test (CAT), and short form to assess depressive symptoms experienced by individuals with SCI, transform scores to the Patient Reported Outcomes Measurement Information System (PROMIS) metric, and create a crosswalk to the Patient Health Questionnaire (PHQ)-9.

**Design:**

We used grounded-theory based qualitative item development methods, large-scale item calibration field testing, confirmatory factor analysis, item response theory (IRT) analyses, and statistical linking techniques to transform scores to a PROMIS metric and to provide a crosswalk with the PHQ-9.

**Setting:**

Five SCI Model System centers and one Department of Veterans Affairs medical center in the United States.

**Participants:**

Adults with traumatic SCI.

**Main Outcome Measures:**

Spinal Cord Injury – Quality of Life (SCI-QOL) Depression Item Bank

**Results:**

Individuals with SCI were involved in all phases of SCI-QOL development. A sample of 716 individuals with traumatic SCI completed 35 items assessing depression, 18 of which were PROMIS items. After removing 7 non-PROMIS items, factor analyses confirmed a unidimensional pool of items. We used a graded response IRT model to estimate slopes and thresholds for the 28 retained items. The SCI-QOL Depression measure correlated 0.76 with the PHQ-9.

**Conclusions:**

The SCI-QOL Depression item bank provides a reliable and sensitive measure of depressive symptoms with scores reported in terms of general population norms. We provide a crosswalk to the PHQ-9 to facilitate comparisons between measures. The item bank may be administered as a CAT or as a short form and is suitable for research and clinical applications.

## Introduction

Depression is one of the most widely studied psychological experiences of persons with spinal cord injury (SCI) due to the high prevalence as well as the burden of illness and disability associated with this condition. In a recent meta-analysis of major depressive disorder (MDD) rates in SCI, Williams and Murray^1^ estimated a point prevalence of 22.2%. This rate is more than three-times higher than the one year prevalence of 6.6% in the general US population.^2^ A slightly higher prevalence of depression diagnosis (28%) was found in United States veterans with SCI.^3^ Depression is also associated with a host of negative outcomes after SCI including urinary tract infections and pressure ulcers,^4^ poorer community mobility and participation,^5,6^ greater unemployment,^7^ and greater risk of mortality.^8^ The high rate of depressive symptoms and associated adverse outcomes is evidence of the need for research on depression assessment and treatment for persons with SCI.

Clinically sensitive depression measures are needed for mood disorder screening. Most depression measures do not use current diagnostic criteria, such as the Diagnostic and Statistical Manual of Mental Disorders criteria.^9^ Only a few studies have examined the validity of a depression screening measure compared to a structured diagnostic interview for persons with SCI^10,11^; in these studies, only one instrument, the Patient Health Questionnaire-9 (PHQ-9),^12,13^ has demonstrated adequate diagnostic accuracy for MDD.^14^ Using a cutoff of 11 or higher, the PHQ-9 has a sensitivity of 100% and a specificity of 84% compared with the Structured Clinical Interview for DSM-IV^15^ diagnosis of MDD during acute SCI rehabilitation.^16^

Two systematic reviews on depression after SCI^10,11^ highlight the paucity of psychometric information of measures. These reviews highlight the variability of diagnostic criteria, symptoms, and time periods of assessment^11^; multidimensionality of commonly-used measures; few validation studies; and poor reporting of psychometric properties.^10^ Neither review recommended one depression measure; scale selection should be guided by the purpose of assessment and clinical considerations. Kalpakjian *et al.*^10^ recommended development of symptom clusters and trajectories and the use of contemporary test development methods. Previous work has shown that depressive symptoms can assume various trajectories after SCI and have typically been identified as chronically high,^17^ improving,^18,19^ worsening,^17,18^ or low.^17^

A conceptual problem with most measures of depressive symptoms in persons with SCI is the inclusion of both somatic (i.e. neurovegetative) and cognitive-affective symptoms. Neurovegetative symptoms overlap with and are likely confounded by the effects of SCI.^10^ Factor analytic studies of depression measures typically find that depression measures, including the PHQ-9,^13,20,21^ the Older Adult Health and Mood Questionnaire,^22^ the Zung Self Rated Depression Scale,^23^ and the Inventory to Diagnose Depression,^24,25^ are multidimensional; that is, they actually measure more than one underlying construct. Multidimensionality obscures the interpretation of symptom etiology, severity, and change; a unidimensional measure reduces ambiguity of scores and increases confidence in utilizing scores to inform clinical decision making.^21,26^

Several problems limit use of depression measures. First, all measures of depression were developed for use in the general population and then applied to individuals with SCI. Second, most measures have been developed without patient input during their development. Third, all of the commonly used measures were developed using classical test theory methods rather than contemporary, item response theory (IRT) approaches.^10^ Consequently, measures like the PHQ-9 have acceptable item functioning, but its psychometric properties are not optimal for SCI populations,^27^ especially when the reporting of somatic complaints may be due to physical aspects of SCI and not depression.

The Patient Reported Outcomes Measurement Information System (PROMIS)^28,29^ includes a depression item bank,^30^ which was developed with patient feedback to represent a wide range of symptom severity and to ensure content validity of the items from a patient perspective.^31^ The depression item pool was calibrated using graded response IRT in a large, general population sample (*N *= 14,839).^30^ The item bank contains primarily cognitive-affective items. Pilkonis *et al.* believe that the exclusion of somatic complaints makes the scale useful in medical populations in which physical symptoms can confound depressive symptom measurement.^30^

There are several advantages to IRT-based item banks over measures developed using classical test theory approaches. IRT-developed scales include items that measure symptoms across a wide range of severity. Tests developed with classical test theory methods typically exclude items at the extreme ends of the distribution. Extreme items are dropped due to poor item-total correlations. In the PROMIS Depression bank, for example, the item ‘I felt sad’ is the *least difficult* item to endorse, while ‘I thought about suicide’ is the *most difficult*. Inclusion of a broad spectrum of items results in an item bank that has greater reliability and measurement precision across a wider range of depressive symptoms than classical test theory-based measures.^32^ IRT-based measures allow the use of computer adaptive testing (CAT); items can be administered in a targeted and brief manner while maintaining measurement precision. Administering short form scales with a fixed subsample of items is also facilitated.

The ability to monitor change provides critical information on the natural history of depression and the optimal timing of interventions. It is therefore essential to use depression severity measures that are sensitive to change and developed in a patient-centered manner if the field of SCI rehabilitation is to make progress toward developing effective treatments for depression. The ‘gold standard’ measure of depression severity in pharmacologic treatment trials is the Hamilton Depression Rating Scale (HDRS).^33^ Unfortunately, the HDRS is multidimensional and has limited sensitivity to change.^34,35^ Maier used Rasch analyses to develop a unidimensional measure of depression severity from the HDRS that is more sensitive than the original score.^36^ In treatment trials involving SCI patients, depression measures that include somatic items may not detect improvement.^37^ Indeed, in a recent trial of venlafaxine XR for MDD in people with SCI, a unidimensional subscale of the HDRS detected improvement in depression while the full HDRS scale did not.^38^

The purpose of this report is to describe development of the SCI-QOL Depression item bank and short forms. The item bank was derived largely from the PROMIS scales, but a large SCI sample was used to develop SCI-specific calibrations that ensure items are free from bias and item selection will be optimized for an SCI population. We report the calibration of the SCI-QOL Depression item bank, its psychometric properties, and comparability to the PROMIS Depression item bank. We provide information on how we transformed scores to the PROMIS metric. Because the PHQ-9 is one of the most widely used depression measures with SCI samples, we provide a crosswalk between the SCI-QOL depression item bank and the PHQ-9.

## Methods

### Overview of the sampling plan

As described in Tulsky *et al*. (this issue),^39^ the SCI-QOL study involved several phases with different samples and procedures. The results presented in this manuscript came from (1) focus groups to define measurement domains and develop item pools; (2) field tests to calibrate the item pool and develop an item bank that could be administered via CATs and short forms; and (3) validation with criteria measures at several time points. We describe each sample, the methods, analytic plan, and results.

### Development of a depression item pool

We began by identifying candidate items from our pilot work which included semi-structured interviews and focus groups with patients with SCI and clinicians with SCI experience (see Tulsky *et al*.^40^ for a full description). Comments from individual interviews formed the initial 38 items in the pool, while focus group feedback yielded an additional 68 ‘new’ items. We selected 27 items, verbatim, from the Neuro-QOL measurement system, 18 of which were also verbatim PROMIS items. Four of those items were subsequently deleted from Neuro-QOL but we retained them in our preliminary item pool. Many of the new items created from interviews and focus groups were redundant with the established Neuro-QOL/PROMIS items. In these cases, if the overlap was deemed sufficient, we dropped the new items in favor of those from Neuro-QOL/PROMIS.

Next, the preliminary item set underwent expert item review,^41^ a method whereby co-investigators reviewed items for relevance and clarity, and made suggestions for revisions and deletions. We arranged items hierarchically to reflect symptom severity. Team members removed redundant items where there was oversaturation in the middle range of the hierarchy, and suggested new items to fill gaps in content coverage. Throughout the process, whenever a new item was redundant with a Neuro-QOL/PROMIS item, we retained the existing (Neuro-QOL/PROMIS) items.

For all newly written items, we asked persons with SCI to answer each item and describe the process they used to select a response. This procedure, called cognitive debriefing,^42^ in which respondents were asked to answer each item, then describe the process they used to come up with their answer and relate whether they perceived anything to be confusing, unclear, or derogatory, or whether they thought any items could be better phrased. For this item pool, we did not need to modify any items based on cognitive interview feedback. We reviewed the 8 remaining ‘new’ items for translatability^43^; none of the items required modification. Note that items from Neuro-QOL and/or PROMIS had already undergone this level of review during their parent project so they were excluded from the cognitive debriefing interviews as well as the translatability review process. A final step was to review the reading level of the item pool using the Lexile framework^44^; all items were written at or below a 5^th^ grade reading level. The final pool for field testing consisted of 35 items, 23 of which were final Neuro-QOL items (18 of these were also from PROMIS), 4 of which were former Neuro-QOL items, and 7 of which were newly written during the item development phase of this project.

### Item calibration and PHQ crosswalk procedures

We recruited 716 subjects as a part of a large-scale, multisite item calibration study from the Kessler Foundation, University of Michigan, Rehabilitation Institute of Chicago, University of Washington, Craig Hospital, and the James J. Peters/Bronx Veterans Administration hospital. Inclusion criteria were age 18 years and older, ability to read and understand English, and medically-documented traumatic SCI. We stratified the sample by level (paraplegia versus tetraplegia), completeness of injury (complete vs. incomplete), and time since injury (<1 year, 1–3 years, and >3 years) to obtain a heterogeneous sample. Neurologic level was documented by the most recent American Spinal Injury Association Impairment Scale (AIS) rating.^45^ Subjects completed the items in a structured interview in person or by telephone. Tulsky *et al*. describes the methods in detail.^46^ A subset of the sample completed the PHQ-9 items during the same testing session.

### Reliability sample and data collection procedures

An independent sample of 245 individuals at the University of Michigan, Kessler Institute for Rehabilitation, Rehabilitation Institute of Chicago, and Craig Hospital completed the item banks twice as part of a larger study.^46^ Each site's Institutional Review Board reviewed and approved the study protocol. Eligibility criteria were similar to the calibration study: traumatic SCI, 18 years or older, and ability to read, speak, and understand English fluently. We stratified the sample by level and completeness of injury as well as time since injury (≤2 years, >2 years). Participants were community-dwelling and sustained SCI more than 4 months before the assessment.

### Item calibration

Item calibration involved confirmation of construct unidimensionality, use of a graded-response IRT model to calibrate item parameters (slopes and thresholds), and examination of differential item functioning (DIF). We used confirmatory factor analyses (CFA) to determine if items conformed to a unidimensional model. Acceptable model fit indices were: CFI >0.90, RMSEA < 0.08, good; CFI > 0.95, RMSEA< 0.06, excellent. We removed items that demonstrated local item dependence (LID; residual correlation >| 0.2|), significant (P < 0.05), misfit (S-X^2^ test),^47^ or DIF^48^ due to sex, age (<50 vs. ≥50), education (some college or less vs. college degree or higher), injury level (paraplegia vs. tetraplegia), severity (complete vs. incomplete), and time post injury (<1 year vs. ≥1 year). We ran the graded response IRT analyses iteratively and removed poorly fitting items. Once we achieved a unidimensional model, we used the IRT parameters to develop CAT algorithms for the item bank. We programmed the CAT in the NIH Assessment Center (http://www.assessmentcenter.net) and selected items for a short form which can also be downloaded as a PDF from the Assessment Center website. Tulsky *et al.*^46^ within this special issue described the detailed methodology and data analysis plan.

### Reliability analysis

To assess test-retest reliability, we calculated Pearson’s *r* and the intraclass correlation coefficient (ICC) with data from the baseline and 1–2 week retest assessments.

### Transformation to PROMIS metric

We computed a linear transformation of SCI-QOL Depression item parameters and scores so that scores reference PROMIS’ general population metric. Thus, a SCI-QOL Depression score of 50 represents the mean of the general population rather than the mean of the SCI sample. The transformation procedure consisted of 6 steps.^46^ First, we used counts of SCI-QOL calibrations and anchor items common to PROMIS and SCI-QOL to determine the linking configuration. We identified IRT parameters for anchor items, then used the Stocking-Lord method^49^ to identify the transformation coefficients to link items. For the anchor items, we examined item-response plots and scatter plots of item parameters, estimated transformation constants, and transformed the item parameters accordingly.

### Crosswalk to PHQ-9

We created a crosswalk from the SCI-QOL Depression item bank to the PHQ-9^50^ so that PHQ-9 raw summed scores have a corresponding SCI-QOL T-score, which allows for direct comparison of the SCI-QOL Depression with PHQ-9 scores. We used the linking methodology and analytic procedures that were developed for the PROsetta Stone project.^51^

## Results

### Participant characteristics of samples

Demographic and injury characteristics of the calibration and PHQ Crosswalk samples are presented in Table 1. Tulsky *et al*.^46^ provides additional details on the focus group and reliability samples.

**Table 1:** Demographic and Injury Characteristics of Calibration Sample and PHQ Crosswalk Subsample

Variable	Calibration Sample (*N* = 716) Mean (SD), N (%)	PHQ Crosswalk Subsample (*n* = 465)
Age (years)	43.0 (15.3)	41.8 (15.6)
Sex		
Male	558 (78%)	363 (78%)
Female	158 (22%)	102 (22%)
Ethnicity		
Hispanic	81 (11%)	57 (12%)
Non-Hispanic	631 (88%)	405 (87%)
Not provided (refused)	4 (1%)	3 (1%)
Race		
Caucasian	505 (70%)	319 (68.6%)
African-American	125 (17%)	90 (19.4%)
Asian	8 (1%)	5 (1.1%)
More than one race	9 (1%)	8 (1.7%)
Other	56 (8%)	39 (8.3%)
Not Provided (refused)	13 (2%)	4 (0.9%)
Time Since Injury	7.1 (10.0)	6.56 (9.5)
<1 year post injury	195 (27%)	161 (34.6%)
1–3 years post injury	186 (26%)	118 (25.4%)
>3 years post injury	335 (47%)	186 (40.0%)
Diagnosis		
Paraplegia Complete	182 (25%)	128 (27%)
Paraplegia Incomplete	143 (20%)	90 (19%)
Tetraplegia Complete	157 (22%)	98 (22%)
Tetraplegia Incomplete	230 (32%)	147 (32%)
Unknown/Missing	2 (0%)	2 (0.4%)

### Preliminary analysis and item removal

Following the first round of analyses on the initial 35-item pool, 5 items were removed. Three of the removed items were Neuro-QOL items that had been included with slightly incorrect wording (e.g. ‘I felt lonely even when I *am* with other people’ instead of ‘I felt lonely even when I *was* with other people). Fortunately, we had also included the correctly-worded version of each of these three items, so we removed the incorrectly worded version of each from the pool. The other 2 items were removed for LID and misfit (significant S-X^2^ test), respectively. Analyses were repeated on the 30-item pool, and an additional 2 items were removed due to LID (both items) and DIF for sex (the item NQDEP09, ‘I felt like crying’). For the final 28-item set, internal consistency was α = 0.964 and item/total correlations ranged from 0.51 to 0.81. For 26 of the items, over 30% of the sample selected category 1 (Never). No item had sparse data (i.e. <5 responses) in any category. Two items had a category inversion where the average raw score (for all items) for persons selecting category ‘5’ (Always) was lower than the average for person selecting category ‘4’ (Often).

### Dimensionality

We observed a unidimensional model (CFI = 0.968; RMSEA = 0.066). Twenty-six items had *R*^2^ values greater than 0.40, and 2 items were less than 0.40. We identified no local dependence, defined as residual correlations >|0.20|. The ratio of the first to second eigenvalue was 14.3.

### IRT parameter estimation and model fit

Slopes (item discrimination parameters) ranged from 1.43 to 4.36; thresholds (item difficulty parameters) ranged from –0.87 to 2.84.

The measurement precision in the theta range between –0.7 and 2.8 is roughly equivalent to a classical reliability of 0.95 or better.

We examined the S-X^2^ model fit statistics using the IRTFIT macro program.^55^ All items had adequate or better model fit statistics (P < 0.05), with marginal reliability equal to 0.950.

### Differential item functioning

We used *lordif*^48^ to examine differential item functioning for age (≤49 vs. ≥50 years), sex (male vs. female), education (some college and lower vs. college degree and above), diagnosis (tetraplegia vs. paraplegia), injury severity (incomplete vs. complete), and time post injury (>1 year vs. <1 year). We flagged 10 items for possible DIF with χ^2^ tests’ P < 0.01 and effect sizes (McFadden's pseudo *R*^2^) >0.02, which is a small but non-negligible effect. On examination of effect sizes, all DIF was negligible and we retained all items. Descriptive statistics for the retained item are presented in Table 2.

**Table 2:** SCI-QOL Depression Descriptive Item Statistics (Calibration Sample, *n* = 716**)**

Item ID	Item Stem	Mean	SD	% at Min	% at Max
Dep_31	I had given up on my dreams.	1.76	1.098	59.9	2.7
**EDDEP04**^PN^	**I felt worthless**.	**1**.**66**	**1**.**025**	**63**.**6**	**2**.**5**
**EDDEP05**^PN^	**I felt that I had nothing to look forward to**.	**1**.**66**	**1**.**017**	**63**.**1**	**2**.**2**
EDDEP06^PN^	I felt helpless.	1.99	1.155	48.0	4.5
EDDEP07^PN^	I withdrew from other people.	1.72	0.973	56.6	1.1
**EDDEP09**^PN^	**I felt that nothing could cheer me up**.	**1**.**73**	**0**.**988**	**56**.**7**	**1**.**7**
EDDEP17^PN^	I felt sad.	2.38	1.122	27.0	4.7
EDDEP19^PN^	I felt that I wanted to give up on everything.	1.42	0.853	75.8	1.3
EDDEP28^PN^	I felt lonely.	2.24	1.184	37.2	4.1
**EDDEP29**^PN^	**I felt depressed**.	**2**.**04**	**1**.**112**	**43**.**3**	**3**.**4**
EDDEP31^PN^	I felt discouraged about the future.	2.16	1.139	39.1	3.4
EDDEP35^PN^	I found that things in my life were overwhelming.	2.05	1.085	41.3	3.4
**EDDEP36**^PN^	**I felt unhappy**.	**2**.**22**	**1**.**117**	**33**.**8**	**3**.**8**
**EDDEP39**^PN^	**I felt I had no reason for living**.	**1**.**35**	**0**.**805**	**79**.**3**	**1**.**4**
**EDDEP41**^PN^	**I felt hopeless**.	**1**.**65**	**0**.**987**	**63**.**0**	**2**.**0**
**EDDEP45**^PN^	**I felt that nothing was interesting**.	**1**.**78**	**1**.**022**	**54**.**6**	**2**.**1**
EDDEP46^PN^	I felt pessimistic.	2.01	1.073	43.4	2.2
**EDDEP48**^PN^	**I felt that my life was empty**.	**1**.**78**	**1**.**055**	**55**.**9**	**2**.**5**
EDDEP54^PN^	I felt emotionally exhausted.	2.09	1.127	42.0	3.6
NQDEP01	I felt lonely even when I was with other people.	1.74	1.000	57.4	1.8
NQDEP08^N^	I was critical of myself for my mistakes.	2.47	1.242	30.3	7.0
NQDEP15	I wished I were dead and away from it all.	1.34	0.832	81.8	1.7
**NQDEP16**	**I thought about suicide**.	**1**.**20**	**0**.**625**	**88**.**5**	.**8**
NQDEP20^N^	I felt unloved.	1.53	0.931	69.0	2.1
NQDEP22	I felt that others would be better off if I were dead.	1.40	0.890	78.4	2.4
NQDEP26^N^	I had trouble keeping my mind on what I was doing.	2.09	1.058	38.4	2.0
NQDEP29^N^	I felt like I needed help for my depression.	1.66	0.997	62.4	1.8
NQDEP30^N^	I had trouble enjoying things that I used to enjoy.	2.40	1.255	34.0	7.1

^P^PROMIS Item.

^N^Neuro-QOL Item.

*Context for all items was: ‘In the past 7 days…’.

Response set was: 1 = Never/2 = Rarely/3 = Sometimes/4 = Often/5 = Always.

**Bold text** indicates items selected for the short form 10a. SCI-QOL Items and parameters copyright © 2015 David Tulsky and Kessler Foundation. All Rights Reserved. Neuro-QOL items © David Cella. PROMIS items © Promis Health Organization. All Rights reserved. Scales should be accessed and used through the corresponding author or http://www.assessmentcenter.net. Do not modify items without permission from the copyright holder.

### Transformation to PROMIS metric

The SCI-specific calibrations are based on the calibration sample. We transformed these SCI-QOL measures to PROMIS’ general population norms. We calculated the transformation constants, slope and intercept, for the 18 PROMIS items using Stocking-Lord techniques^49^ and applied them to create linear transformations for each SCI-QOL parameter. Thus, SCI-QOL scores are reported as a PROMIS Depression score with higher scores indicating more severe depressive symptoms. Transformed slopes range from 1.39 to 4.23, and thresholds range from –0.677 to 3.143 (Table 3). With CAT administration, the Assessment Center automatically transforms IRT-based scaled scores into T-scores with a mean of 50 and SD of 10 (Table 4).

**Table 3:** SCI-QOL Depression Items and IRT Parameters (Calibration Sample, *N* = 716**)**

Item ID	Item Response Theory Calibration Statistics					
	Item Stem	Slope	Threshold 1	Threshold 2	Threshold 3	Threshold 4
Dep_31	I had given up on my dreams.	2.15998	0.41389	1.02506	1.69092	2.54440
**EDDEP04**^PN^	**I felt worthless.**	**3**.**55165**	**0**.**47556**	**0**.**93740**	**1**.**70107**	**2**.**18427**
**EDDEP05**^PN^	**I felt that I had nothing to look forward to.**	**4**.**23071**	**0**.**44395**	**0**.**93198**	**1**.**65946**	**2**.**14865**
EDDEP06^PN^	I felt helpless.	2.32652	0.05125	0.66820	1.62690	2.18065
EDDEP07^PN^	I withdrew from other people.	2.28323	0.30366	1.01594	1.97905	2.98945
**EDDEP09**^PN^	**I felt that nothing could cheer me up.**	**3**.**27472**	**0**.**28513**	**0**.**93234**	**1**.**79038**	**2**.**35423**
EDDEP17^PN^	I felt sad.	2.82139	−0.60395	0.22965	1.27734	1.98449
EDDEP19^PN^	I felt that I wanted to give up on everything.	3.43829	0.83163	1.32431	1.98523	2.56590
EDDEP28^PN^	I felt lonely.	1.77956	−0.33732	0.38464	1.45255	2.49997
**EDDEP29**^PN^	**I felt depressed.**	**3**.**40087**	−**0**.**07614**	**0**.**52751**	**1**.**47203**	**2**.**06163**
EDDEP31^PN^	I felt discouraged about the future.	2.77590	−0.19851	0.42318	1.39802	2.18645
EDDEP35^PN^	I found that things in my life were overwhelming.	2.15291	−0.15561	0.62625	1.82065	2.40451
**EDDEP36**^PN^	**I felt unhappy.**	**3**.**28354**	−**0**.**35504**	**0**.**38967**	**1**.**34430**	**2**.**02462**
**EDDEP39**^PN^	**I felt I had no reason for living.**	**3**.**39440**	**0**.**96514**	**1**.**48114**	**2**.**01474**	**2**.**49881**
**EDDEP41**^PN^	**I felt hopeless.**	**4**.**22822**	**0**.**44127**	**0**.**96638**	**1**.**71221**	**2**.**21806**
**EDDEP45**^PN^	**I felt that nothing was interesting.**	**2**.**52519**	**0**.**24102**	**0**.**89763**	**1**.**84310**	**2**.**48422**
EDDEP46^PN^	I felt pessimistic.	2.11924	−0.10251	0.64033	1.79079	2.67257
**EDDEP48**^PN^	**I felt that my life was empty.**	**3**.**02589**	**0**.**26893**	**0**.**91047**	**1**.**64966**	**2**.**28172**
EDDEP54^PN^	I felt emotionally exhausted.	2.09932	−0.14714	0.52312	1.68354	2.37133
NQDEP01	I felt lonely even when I was with other people.	2.64925	0.30872	0.89880	1.88422	2.55140
NQDEP08^N^	I was critical of myself for my mistakes.	1.39097	−0.67682	0.14277	1.41130	2.44408
NQDEP15	I wished I were dead and away from it all.	3.13286	1.08723	1.48687	2.01529	2.45718
**NQDEP16**	**I thought about suicide.**	**2**.**59718**	**1**.**46551**	**1**.**89301**	**2**.**56600**	**2**.**97717**
NQDEP20^N^	I felt unloved.	1.89448	0.72376	1.40645	2.34282	2.86678
NQDEP22	I felt that others would be better off if I were dead.	2.76672	0.96544	1.42641	2.00417	2.36611
NQDEP26^N^	I had trouble keeping my mind on what I was doing.	1.59980	−0.28470	0.63940	1.96753	3.14329
NQDEP29^N^	I felt like I needed help for my depression.	2.19849	0.48058	1.08175	1.96821	2.70108
NQDEP30^N^	I had trouble enjoying things that I used to enjoy.	1.92691	−0.41660	0.20044	1.27276	2.02554

^P^PROMIS Item.

^N^Neuro-QOL Item.

*Context for all items was: ‘In the past 7 days…’.

Response set was: 1=Never/2=Rarely/3=Sometimes/4=Often/5=Always.

**Bold text** indicates items selected for the short form 10a. SCI-QOL Items and parameters copyright © 2015 David Tulsky and Kessler Foundation. All Rights Reserved. Neuro-QOL items © David Cella. PROMIS items © Promis Health Organization. All Rights reserved. Scales should be accessed and used through the corresponding author or http://www.assessmentcenter.net. Do not modify items without permission from the copyright holder.

**Table 4:** Linking Table: SCI-QOL Depression and PHQ-9

PHQ- 9	SCI-QOL	SE
*Raw Score*	*T-Score*	
9*	22	9.85
10	46	3.50
11	48	2.50
12	49	2.00
13	50	1.70
14	51	1.48
15	52	1.32
16	53	1.19
17	53	1.09
18	54	1.00
19	55	0.92
20	55	0.86
21	56	0.80
22	56	0.75
23	57	0.71
24	58	0.67
25	58	0.63
26	59	0.59
27	60	0.56
28	60	0.54
29	61	0.51
30	62	0.49
31	63	0.47
32	63	0.45
33	64	0.43
34	65	0.41
35	66	0.40
36	67	0.38

*Note: PHQ items were scored 1–4.

### Short form selection and mode of administration

We programmed item parameters into the NIH Assessment Center^SM^^53^ to facilitate CAT administration. Users can modify configurations to maximize reliability or reduce test burden, or select specific items. A short form is also available.

SCI-QOL uses PROMIS’ default discontinue criteria; the minimum number of items is four and the maximum is 12 with a maximum standard error of 0.3. Thus, the CAT always administers at least 4 items and will discontinue when the standard error of a score estimate drops below 0.3 or 12 items are administered. Users may change the discontinue criteria so that additional items are administered when a more precise assessment is needed. For instance, if the user selects an option that the CAT administers a minimum of 8 items before discontinuing, a lengthier test will be administered, but a more reliable score will be obtained.

In situations where it may not be feasible to use a laptop or tablet computer with internet access, users may want to use a short form. We developed a 10-item short form with the goal of including the most informative items across a wide range of depressive symptoms. The items selected for this form, the SCI-QOL Depression short form 10a, are indicated by bold text in Tables 2 and 3. Since items are calibrated on a common metric, short form scores are comparable to those obtained from a CAT or full item bank. Investigators and clinicians can develop custom short forms which can be scored on the same metric. Short forms are scored by summing the item responses and finding the associated T-score in Table 5.

**Table 5:** T-score lookup table for SCI-QOL Depression Short Form 10a

RAW SCORE	T-SCORE	STANDARD ERROR
10	38.3	5.8
11	44.6	3.5
12	47.1	3.1
13	48.9	2.7
14	50.4	2.4
15	51.6	2.2
16	52.7	2.1
17	53.7	2.0
18	54.6	2.0
19	55.4	1.9
20	56.3	1.9
21	57.0	1.9
22	57.8	1.9
23	58.6	1.9
24	59.3	1.9
25	60.1	1.9
26	60.8	1.9
27	61.5	1.9
28	62.2	1.9
29	62.9	1.9
30	63.6	1.9
31	64.3	1.9
32	65.0	1.9
33	65.7	1.8
34	66.3	1.8
35	67.0	1.8
36	67.6	1.8
37	68.3	1.8
38	68.9	1.8
39	69.6	1.8
40	70.3	1.8
41	71.0	1.9
42	71.7	1.9
43	72.5	2.0
44	73.3	2.0
45	74.2	2.1
46	75.2	2.3
47	76.2	2.4
48	77.6	2.7
49	79.0	2.9
50	81.9	3.7

We evaluated measurement precision of the full bank, 10-item short form, and variable-length CAT with the default minimum of 4 items. Table 6 presents the mean, standard deviation, range, and standard error ranges for these administration modes; Fig. 2 presents the associated reliability curves.[Fig F1]

**Figure 1 F1:**
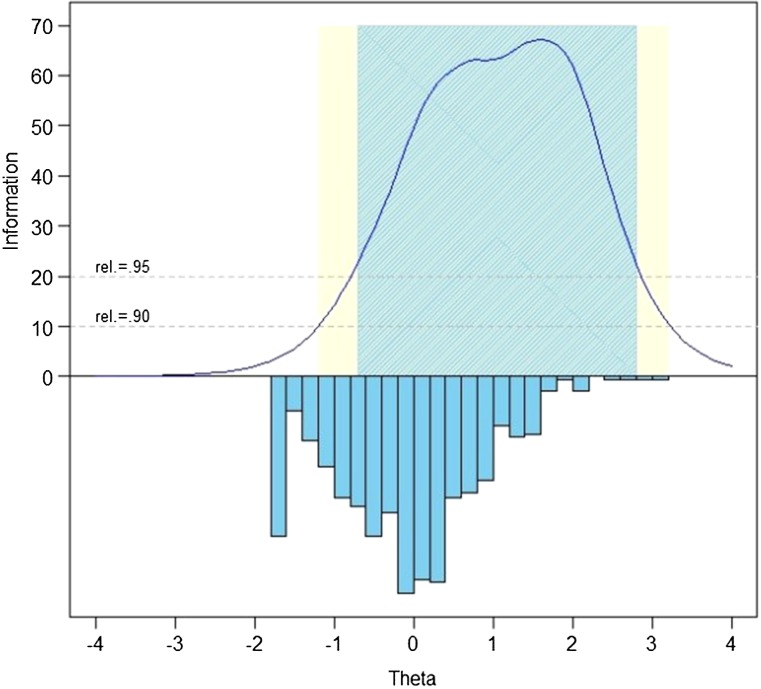
SCI-QOL Depression Item Bank Information and Precision.

**Figure 2:**
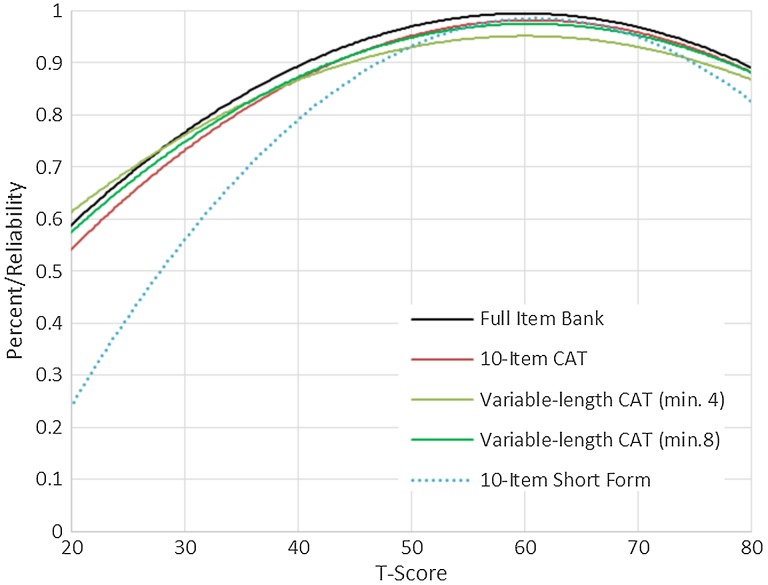
SCI-QOL Depression: Reliability by T-score and Assessment Method.

**Table 6:** Breadth of Coverage for SCI-QOL Depression, PHQ-9, and PHQ-2 (Calibration and PHQ Crosswalk Samples)

Measure/Mode		# Items Admin				Score				
		Mean(SD)	Min	Max	Mean(SD)	Range	% Floor	% Ceiling	SE	Reliability
**SCI-QOL Depression**	Full Bank	28(0)	28	28	50.(9.6)	32.28–83.86	0.1%	3.1%	2.1	94.97%
	8-Item fixed length CAT	8(0)	8	8	50.7(9.4)	34.11–83.41	4.7%	0.1%	2.7	92.40%
	Variable-Length CAT (min 4/max 12)	6.41(3.3)	4	12	50.7(9.5)	32.84–82.56	3.5%	0.1%	3.0	91.45%
	Variable-Length CAT (min 4/max 8)	5.45(1.8)	4	8	50.7(9.4)	34.11–83.41	4.7%	0.1%	3.1	90.87%
	4-item Fixed-Length CAT	4(0)	4	4	50.7(9.1)	37.27–80.17	7.4%	0.1%	3.4	88.87%
**PHQ-9**		9(0)	9	9	48.7(8.7)	35.77–76.89	10.2%	0.2%	4.4	83.39%
**PHQ-2**		2(0)	2	2	49.0 (7.6)	42.08–69.84	46.0%	3.2%	5.8	74.54%

*Note: Sample mean values are reported for SE and Reliability, respectively.

### Reliability

We used the default stopping rules for the CAT: minimum of 4 and maximum of 12 items with the community sample. Administration averaged 5.93 items (SD 3.1); 75% of the sample completed the CAT within 6 items, and 17.7% received the maximum number of items (12). When comparing SCI-QOL Depression scores at baseline with those from the 1–2 week follow up assessment (*n* = 245), Pearson’s *r* = 0.80 (P < .001) and ICC (2,1) = 0.80 (95% CI: 0.75 to 0.84).

### Crosswalk to PHQ-9

**Figure 3:**
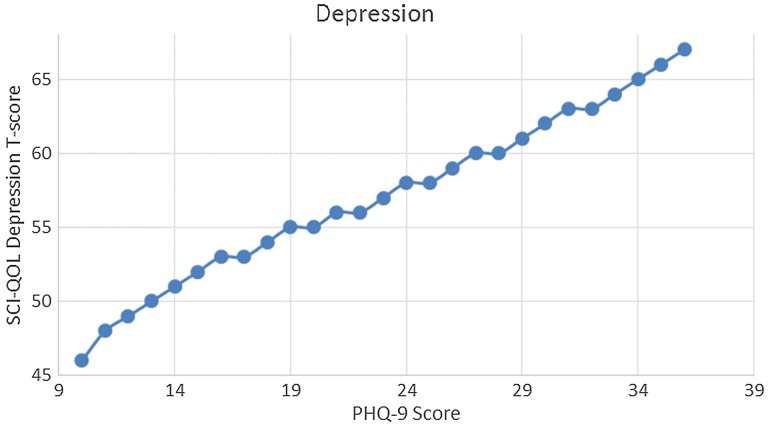
Relationship Between SCI-QOL Depression T-scores and PHQ-9 Raw Scores.

**Figure 4:**
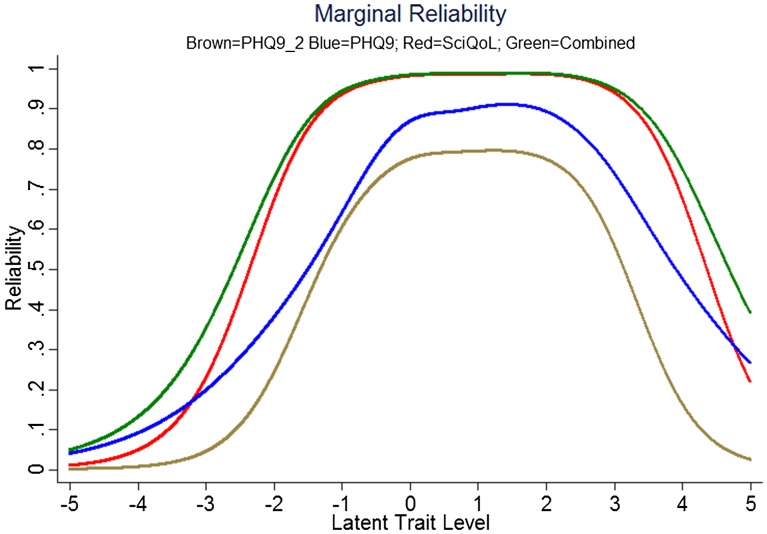
Marginal Reliability of PHQ-2, PHQ-9, SCI-QOL Depression, and Combined (*n *= 465). Colors relate to the online version of the figure.

**Figure 5:**
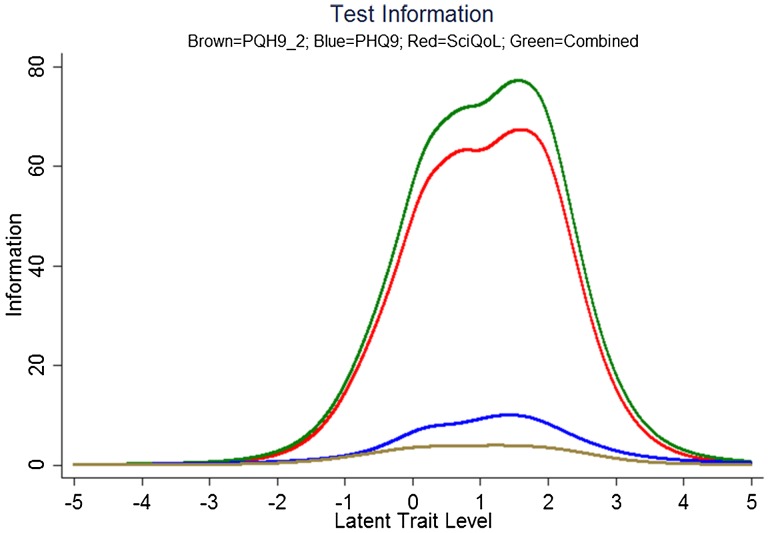
Scale information provided by the PHQ-2, PHQ-9, SCI-QOL Depression, and Combined. Note: Scale information is shown on the y-axis and T-scores are shown on the X-axis. The scale (or test) information curve (or function) indicates the level of information (i.e. reliability) provided by the scale over the range of the construct continuum. Colors relate to the online version of the figure.

We produced a crosswalk from the SCI-QOL Depression item bank to the PHQ-9 using a similar linking procedure as was conducted by Gershon *et al*.^54,55^ with data from a general population sample collected as part of the NIH Toolbox. Fig. 3 displays the relationship between SCI-QOL Depression and the PHQ-9 in our sample; the correlation was 0.76. Fig. 4 demonstrates the superior marginal reliability of the SCI-QOL items (or the SCI-QOL + PHQ-9 items) when compared to the PHQ-9 or PHQ-2 (which includes only the first 2 items of the PHQ for a very brief screening). Fig. 5 demonstrates the test information that is conveyed by the measures. Test information indicates the precision of measurement provided by the item bank across different scores; that is, the more information a test has, the more accurately it can determine what level of an underlying trait (in this case, depression) a given participant possesses. Test information has an inverse relationship with error variance; the more information a test has, the smaller the error of measurement. The figure shows the scale information of the SCI-QOL Depression bank, the PHQ-9, and the PHQ-2, as well as a combined score (including all of the SCI-QOL *and* PHQ-9 items) across the range of depression. The SCI-QOL provides greater scale information than the PHQ-9 or the PHQ-2 across individuals with scores ranging from 2 standard deviations below the mean through 4 standard deviations above the mean. As expected, the combined item bank yields the most information since it uses all component items (SCI-QOL and PHQ).These values have been used to generate the PHQ-9 raw score to SCI-QOL T-Score metric conversion crosswalk table (Table 4).

As indicated by the correlation between measures (*r* = 0.76), scores on the SCI-QOL Depression bank and the PHQ-9 are not strictly interchangeable at the *individual* level. For the subsample completing the item bank and PHQ-9, we used the PHQ-9 to estimate an expected SCI-QOL score and then calculated a discrepancy score by subtracting the observed value from the predicted score. The predicted and observed scores were within a half of a standard deviation for over half the sample. However, there was a substantial number of people (*n* = 106) who had discrepancies greater than 1 SD (this is consistent with the shared variance indicated by the correlation between the measures).

## Discussion

The goal of this study was to (1) develop a bank of depression-related items for use with individuals with SCI; (2) evaluate the psychometric properties of the item bank; (3) develop a cross-walk from the PHQ-9 to SCI-QOL; and (4) provide information that facilitates clinical and research use of the depression item bank.

The SCI-QOL Depression bank is an optimized version of the PROMIS v1.0 Depression item bank for individuals with SCI. Patient and clinician focus group participants confirmed that the PROMIS v1.0 Depression items had content validity in an SCI population. Like the PROMIS Depression bank, the SCI-QOL Depression item bank does not include items related to somatic symptoms which might be confounded with physical medical issues experienced by persons with SCI. We then developed SCI-specific item calibrations using a large, heterogeneous sample of individuals with SCI using a 2-PL graded-response IRT model. We removed items exhibiting DIF, poor item fit statistics, or local dependence. This procedure ensures that the SCI-QOL Depression bank is relevant and appropriate for individuals with SCI. We used IRT linking methods to transform the SCI-QOL calibrations to the PROMIS metric, thus allowing use of SCI calibrations that can be directly compared to PROMIS scores.

The SCI-QOL also provides the end user with several administration options depending upon the intended use of the scale. For studies requiring rapid, quick screening, the SCI-QOL could be administered using CAT stopping rules to reduce testing time (e.g. administer only 4 items regardless of standard error variance). For studies requiring more administration precision and participant burden is not an issue, the CAT stopping rules could be set to administer more items (e.g. a minimum of 8 items, standard error of 0.30, and a maximum number of 12 or more items). When a computer and/or internet connection is not available for testing, a short form could be administered to the participant. Finally, if a special subpopulation is being tested, a customized short form could be developed that only includes items relevant to the subpopulation (e.g. a short form including only the most ‘difficult’ items—i.e. those that will be endorsed only by individuals with the most severe depressive symptomatology—could be created for use in a study of individuals with SCI and concomitant MDD). While administration of the full item bank would yield the highest reliability, use of the full bank is not recommended given the high reliability of the 10-item fixed-length CAT and the variable-length CAT with a minimum of 8 items. Either of these administrations would very closely approximate the scores obtained when a full bank is administered.

The wide use of the PHQ-9 in SCI research studies led us to link SCI-QOL with the PHQ-9. We co-administered the SCI-QOL with the PHQ-9 allowing us to compare the psychometric properties of the instruments as well as developing a crosswalk between the scales. The reliability of the SCI-QOL item bank is superior to the PHQ-9 (or PHQ-2) over a wider range of depressive functioning suggesting that the SCI-QOL has greater measurement precision and is better able to assess individuals at both tails of the distribution. Additionally, the SCI-QOL provides significantly greater scale information than the PHQ-9 or the PHQ-2 which also indicates that the SCI-QOL score is a more reliable measure across the entire range of depressive symptoms, providing more accuracy and sensitivity across a wider range of depression. The SCI-QOL is able to estimate the score for an individual who is one or two standard deviations below the mean and up to three or four standard deviations above the mean, which is far more precise than either the PHQ-9 or PHQ-2. Collectively, these data suggest that the SCI-QOL has greater measurement precision.

We developed a crosswalk table to enable researchers and clinicians who utilize the PHQ-9 to transition to data collection with the SCI-QOL Depression bank. Utilizing Table 4, the PHQ-9 scores can be transformed to a SCI-QOL Depression T-Score metric allowing direct comparison of SCI-QOL to PHQ-9 scores. The two measures are correlated 0.76. Investigators can apply the crosswalk conversions with some confidence at the group level because the majority of cases have small differences between the observed and linked mean scores. At the same time, investigators and clinicians should exercise caution when applying the crosswalks to individuals because the 95% confidence interval is 1.2 SD, making it difficult to predict any individual case with exact precision. Therefore, investigators should use caution in the inferences drawn when using the crosswalk table to track performance of a specific individual over time. Nevertheless, the transformation is useful in answering *sample-level* questions.

### Study limitations

We recruited the samples from a limited number of SCI Model System facilities and one VA medical center; they may not be representative of all persons with SCI in the United States. Persons who volunteered received a modest honorarium which may have introduced self-selection bias. The above study has not tested the predictive validity of the SCI-QOL to predict individuals who are likely to develop MDD or have other adjustment disorders over time. Future research should be conducted to develop appropriate clinical markers for the SCI-QOL if it is to be used in clinical settings.

## Conclusions

The SCI-QOL Depression item bank is the first scale of depressive symptoms that has been developed specifically for an SCI population. The SCI-QOL is an optimized version of the PROMIS v1.0 item bank and the scores are, for all practical purpose, PROMIS scores. Moreover, it is linked to the PHQ-9 so that PHQ-9 scores can be transformed to SCI-QOL equivalent scores allowing researchers to maintain continuity of measurement in an ongoing longitudinal study.

The SCI-QOL Depression item bank reflects the constellation of symptoms experienced by persons with SCI. It is not designed to provide a diagnosis of major depressive disorder, but rather serve as a population-specific indicator of SCI-related depressive symptoms. The Depression item bank contains 28 items; users have the option of using a 10-item short form or CAT. We removed misfitting items systematically, based on psychometric and clinical criteria.

This new measure offers clinicians and researchers a precise, population-relevant, and flexible method to describe symptoms related to depression. The mixed methods approach assures relevance and patient-centered validity, and strengthens our ability to measure this important phenomenon in persons with SCI. Doing so will enhance our ability to identify critical time points of intervention along the SCI rehabilitation and recovery trajectory.

## Suppliers

*Mplus Statistical Analysis with Latent Variables User's Guide* [computer program]. Version 6. Los Angeles: Muthen & Muthen; 2007.

## Disclaimer statements

**Contributors** All authors have contributed significantly to the design, analysis and writing of this manuscript. The contents represent original work and have not been published elsewhere. No commercial party having a direct financial interest in the results of the research supporting this article has or will confer a benefit upon the authors or upon any organization with which the authors are associated.

**Funding** This study was supported by National Institutes of Health grant numbers 5R01HD054659 (Eunice Kennedy Shriver National Institute of Child's Health and Human Development/National Center on Medical Rehabilitation Research and the National Institute on Neurological Disorders and Stroke) and U01AR057929 (NIH Common Fund/National Institute of Arthritis and Musculoskeletal and Skin Diseases) and by the National Institute on Disability and Rehabilitation Research grant numbers H133N060022, H133N060024, H133N060032, H133N060014, H133N060005, and H133N060027.

**Conflicts of interest** No commercial party having a direct financial interest in the results of the research supporting this article has or will confer a benefit upon the authors or upon any organization with which the authors are associated.

All SCI-QOL items and parameters are © 2015 David Tulsky and Kessler Foundation. All rights reserved. All SCI-QOL items originally from Neuro-QOL are © 2008-2013 David Cella on behalf of the National Institute for Neurological Disorders and Stroke (NINDS). Currently all items are freely available to the public via the Assessment Center platform (http://www.assessmentcenter.net). At present, the purpose of the copyright is to protect the integrity of the tool. There are currently no plans for Dr. Tulsky, Kessler Foundation, or the NINDS to benefit financially from the use of the copyrighted material.

**Ethics approval** The Institutional Review Board at each site reviewed and approved this project.
